# Effect of ATP and Bax on the apoptosis of *Eimeria tenella* host cells

**DOI:** 10.1186/s12917-017-1313-z

**Published:** 2017-12-28

**Authors:** Zhiyong Xu, Mingxue Zheng, Li Zhang, Xuesong Zhang, Yan Zhang, Xiaozhen Cui, Xin Gong, Rou Xi, Rui Bai

**Affiliations:** 1 College of Animal Science and Technology, Shanxi Agricultural University, Taigu, 030801 China; 2 College of Animal Science and Technology, Henan Institute of Science and Technology, Xinxiang, 453003 China

**Keywords:** *Eimeria tenella*, Host cell, Apoptosis, ATP, Bax

## Abstract

**Background:**

*Eimeria tenella* (*E. tenella*) is a species of *Eimeria* that causes haemorrhagic caecal coccidiosis, resulting in major economic losses in the global poultry industry. After *E. tenella* infection, the amount of ATP and Bax in host cells showed highly significant changes. Therefore, it is necessary to investigate the effects of ATP and Bax on the apoptosis of *E. tenella* host cells.

**Results:**

The ATP-treated group and the V5-treated group had higher *E. tenella* infection rates than the untreated group at 24, 48, 72, 96, and 120 h after infection with *E. tenella*. The results of flow cytometry showed that compared with the control group, the mitochondrial permeability transition pore (MPTP) opening in the untreated group was highly significantly increased (*P* < 0.01) at 4, 24, 48, 72, 96, and 120 h. Moreover, results from Hoechst-Annexin V-PI staining and flow cytometry showed that the rates of early apoptosis, late apoptosis, and necrosis in the untreated group were significantly lower (*P* < 0.05) or highly significantly lower (*P* < 0.01) than those of the control group at 4 h, while the rates of early apoptosis, late apoptosis, and necrosis in the untreated group were higher at varying degrees than those in the control group at 24–120 h (*P* < 0.05 or *P* < 0.01). After treatment with ATP and Bax inhibitors, the rates of early apoptosis, late apoptosis, and necrosis, in addition to the MPTP opening in both the ATP-treated and V5-treated groups, were significantly lower (*P* < 0.05) or highly significantly lower (*P* < 0.01) than those in the untreated group.

**Conclusions:**

ATP and Bax play important roles in regulating the apoptosis of *E. tenella* host cells.

**Electronic supplementary material:**

The online version of this article (10.1186/s12917-017-1313-z) contains supplementary material, which is available to authorized users.

## Background

*Eimeria tenella* (*E. tenella*) is the most common species of *Eimeria*, and it parasitises chicken intestinal mucosa epithelial cells. After infection with *E. tenella*, the mortality rates in chickens increase up to 80%, whereas weight and egg production significantly decrease [1, 2]. These conditions result in major economic losses in the global poultry industry [3, 4].

*E. tenella* primarily damages the chicken caecum. The apoptosis rate of duodenal mucosal cells infected with *Eimeria acervulina* reaches the highest at 0.5 and 5 days [5]. *E. tenella*-infected embryo caecal epithelial cells have shown decreased apoptosis at early developmental stages (24 h or less) but have conversely shown increased apoptosis at the middle and late developmental stages (24–120 h) [6]. The degree to which the mitochondrial membrane KATP channel opens has been shown to decrease the apoptosis rate of cells infected with *E. tenella* and promote the development of *E. tenella* in chicken caecum epithelial cells [7]. The mitochondrial apoptotic pathway is regulated by concentration changes in Ca^2+^ outside of *E. tenella* host cells and endoplasmic reticulum Ca^2+^ channels [8]. The amount of apoptosis in intestinal epithelial cells infected with *E. tenella* is consistent with the severity of injury to the mitochondrial structure. These observations indicate a positive correlation of apoptosis in cells infected with *E. tenella* with changes in mitochondrial structure [9].

The mitochondrial permeability transition pore (MPTP), a compound channel composed of multiple proteins, is located between inner and outer mitochondrial membranes. A previous study showed that MPTP is a key node that plays a predominant role in the mitochondrial apoptosis pathway in host cells induced by *E. tenella* [10]. Cyclophilin D has previously been identified as an essential component of the MPTP structure [11]. Other studies have also suggested that MPTP potentially comprises a voltage-dependent anion channel (VDAC) and an adenine nucleotide transporter (ANT) [12]. Adenosine triphosphate (ATP) is the sole supplier of energy in living organisms. To maintain cell metabolic activity, ATP is transported into the cytoplasm via ANT, whereas cytoplasmic ADP is transported to mitochondria via ANT, which provides the raw material for oxidative phosphorylation [13]. During ischaemia and hypoxia, decreased levels of ATP result in increased concentrations of cytoplasmic phosphorus and calcium ions and the production of a large number of superoxides, thus further promoting MPTP opening and eventually leading to cell death [14]. Bcl-2 family proteins can be divided into two categories as follows: pro-apoptotic proteins, such as Bax, Bak, Bad and Bid, and anti-apoptotic proteins, such as Bcl-2 and Bcl-xl. Bax primarily resides in the cytoplasm. Apoptosis stimuli increase BH3 expression, which enhances the effects of Bax and Bak by combining with Bcl-2 and Bcl-xl, further promoting cell apoptosis [15]. A previous study demonstrated that Bax could promote cell apoptosis by combining with VDAC [16].

The caspase-9 inhibitor Z-LEHD-FMK can significantly increase the infection rate of *E. tenella* by inhibiting host cell apoptosis [6]. In animal models, the inhibition of MPTP by either cyclosporin A (CsA) or the genetic ablation of CyP-D provides strong protection from both reperfusion injury and congestive heart failure [17]. Other evidence also suggests that apoptosis can be reversed by anti-apoptotic drugs, which can rescue cells and provide new directions for the protective treatment of an organism by avoiding or controlling harmful processes [18]. The control of host-cell apoptosis had been demonstrated as complementary in the treatment of parasitic diseases [19].

In a recent study, we showed that the Bax amount in *E. tenella* host cells visibly decreased during the early developmental stages of *E. tenella* and, conversely, remarkably increased during the middle and later developmental stages [20]. The ATP content decreased at all developmental stages of *E. tenella* [20]. In the present study, we further investigated the effects of ATP and Bax on the apoptosis of *E. tenella* host cells in vitro by flow cytometry (FC), Hoechst-fluorescein isothiocyanate-conjugated Annexin V-propidium iodide (Hoechst-Annexin V-FITC-PI) staining and primary chick embryo caecum epithelial cell culture techniques. These results can provide a theoretical foundation for studying the mechanism of *E. tenella*-induced apoptosis in host cells and exploring anti-apoptotic adjuvant treatment of *E. tenella* infection in chickens.

## Methods

### Experimental animals

A total of twenty 1-day-old chicks and one hundred 15-day-old specific pathogen-free (**SPF**) chicken embryos were used in the present study and were provided by Beijing Meri Avigon Laboratory Animal Technology Co., Ltd. (Beijing, China). The 1-day-old chicks were raised under strict pathogen-free conditions (Isolator. Temperature and pressure: 1–3 d, 35–36 °C, 25 Pa; 4–7 d, 32–35 °C, 25–35 Pa; 8–14 d: 29–32 °C, 35–45 Pa; 15–21 d, 21–25 °C, 55–75 Pa; 22–30 d, 21–25 °C, and 55–75 Pa. Humidity: 1–10 d, 65–70%; 11–30 d, 60–65%).

### Parasites

The *E. tenella* Shanxi virulent strain (**EtSX01**) used in the present study was obtained from the Laboratory of Veterinary Pathology in the College of Animal Science and Technology (Laboratory of Veterinary Pathology, Shanxi Agricultural University; Taigu, China).

### Preparation of *E. tenella* Sporozoites

*E. tenella* was amplified by passage through twenty 20-day-old SPF chicks previously infected orally with 6000 sporulated *E. tenella* oocysts. The resulting oocysts were obtained from the faeces of chickens at seven to eight days post-infection. After the oocysts were isolated and sporulated, the sporozoites were excysted as previously described [6]. The chicks were euthanized with cervical dislocation under deep Nembutal anaesthesia [45 μg/g of body weight (BW), intraperitoneal injection; Shanghai Chemical Factory, Shanghai, China].

### Primary culture of Chick embryo Caecal cells and parasite infection

One hundred 15-day-old SPF chick embryos (Merial Vital Corp, Beijing, China**)** were euthanized with cervical dislocation under deep Nembutal anaesthesia (45 μg/g of BW) for sample preparation. Chick embryo caecal epithelial cells were collected and cultured as previously described [7]. The caeca were briefly removed and placed in PBS, minced to 1 mm^3^, digested by 50 mg/l thermolysin (Sigma, California, America) at 37 °C for 2 h, rinsed with PBS, and centrifuged at 220 g for 5 min to remove single cells. Prior to plating, based on the adherence speed of each cell, the precipitated dissociated cells were cultivated for 70 min at 41 °C in a humidified, 8% CO_2_-air incubator to remove all other cells, except the caecal epithelial cells in culture medium (DMEM; Sigma, California, America) supplemented with 10% FBS [6]. Non-adherent caecal epithelial cell aggregates were maintained at 41 °C in a humidified incubator with 8% CO_2_. The cells were subsequently plated at a concentration of 2 × 10^5^ live cell aggregates per well onto a 6-well tissue culture plate in culture medium supplemented with 2.5% FBS [6]. Confluent chick embryo caecal epithelial cell monolayers were infected with 4 × 10^5^ freshly excysted *E. tenella* sporozoites per well.

### Experimental protocol

When the adherence rate reached 90%, chick embryo caecal epithelial cells in 6-well tissue culture plates were randomly divided into four experimental groups, as follows: (1) the control group (group C); (2) the untreated group (cells infected with *E. tenella* sporozoites, group T0); (3) the treated group I [cells infected with *E. tenella* sporozoites and treated with 30 μmol of ATP (Sigma, California, America), group T1]; and (4) the treated group II [cells infected with *E. tenella* sporozoites and treated with 150 μmol of V5 (H-VPMLK-OH, Bax-Inhibiting Peptide; Merck, New Jersey, America), group T2]. The cells were collected at 4, 24, 48, 72, 96, and 120 h after infection. In addition, the culture liquid was changed every 48 h. Cells in group T1 that were sampled at 4–24 h were treated with ATP at the time of infection with *E. tenella* sporozoites, whereas the cells sampled at 48–120 h were treated with ATP 24 h prior to sampling. Cells in group T2 that were sampled at 4 h were treated with V5 at the time of infection with *E. tenella* sporozoites, while the cells sampled at 24–120 h were treated with V5 4 h prior to sampling.

### Haematoxylin and eosin stain (HE staining)

At 4, 24, 48, 72, 96, and 120 h after infection, the chamber slides of groups C and T0 were collected and subsequently stained with Lillie-Mayer’s haematoxylin (Solarbio) and 1% eosin (Solarbio) as previously described [6]. Sample *E. tenella* infections were observed in 200 randomly selected cells by light microscopy.

The infection rates at each time point (%) = the number of infected cells at each time point/200 × 100.

### Dynamic detection of MPTP opening in *E. tenella* host cells

Fluorescent Calcein AM (Life Technology, New York, America; Cat.C3099 Lot: 1,311,548) and CoCl_2_ (Sigma, Lot: 232,696 No: BCBG0246V) markers were employed to detect the dynamic changes of MPTP opening using FC. At 4, 24, 48, 72, 96, and 120 h after infection, chick embryo caecal epithelial cells were harvested using 0.25% trypsin, rinsed in PBS, centrifuged at 600 g for 5 min, suspended in 200 μl of binding buffer (Sigma, California, America), and incubated with Calcein AM and CoCl_2_ (15 min, 37 °C, in the dark). Subsequently, 250 μl of binding buffer was added to the cells, and the mixture was subjected to FC analysis. Flow cytometry (American BD, FACSCalibur) was performed as previously described [21]. The results were analysed using CellQuest software. Fluorescent intensity reflects a change in MPTP opening [22].

### Dynamic detection of apoptosis in *E. tenella* host cells

The Annexin V-FITC/PI (Invitrogen, New York, America; Lot: 1,223,786, model: V13241) was used in the present study. The methods used to harvest and incubate the cells were consistent with those used to detect MPTP opening. Subsequently, chick embryo caecal epithelial cells were re-suspended and incubated with 5 μl of Annexin V-FITC and 1 μl of PI for 30 min at room temperature in the dark. Next, the cells were added to 200 μl of binding buffer and subjected to FC analysis. Annexin V-/PI- quadrant: viable cells; Annexin V+/PI- quadrant: early apoptotic cells; Annexin V+/PI+ quadrant: late apoptotic and necrotic cells.

Annexin V-FITC/PI and Hoechst 33,342 (Beyotime, Shanghai, China; cat: C1025) markers were employed to detect the apoptosis rate under a fluorescence microscope (OLYMPUS, Japan). The procedures were performed according to Venkatanarayan [23]. Briefly, at 4, 24, 48, 72, 96, and 120 h after infection, the culture media from groups C and T0 were aspirated into suitable centrifuge tubes. Subsequently, chick embryo caecal epithelial cells were harvested using 0.25% trypsin, centrifuged at 1000 *g* for 5 min, and suspended with 1 ml of ice-cold PBS. The cells were centrifuged at 1000 *g* for 5 min and gently suspended in 400 μl of 1× binding buffer (1 × 10^5^ cell density), to which 5 μl of 2 μg/ml Hoechst 33,342 was added at 37 °C. The mixture was allowed to stand for 15 min, centrifuged at 1000 *g* for 5 min, and gently suspended in 400 μl of 1× binding buffer. Subsequently, 5 μl of Annexin V-FITC was added in the dark at room temperature. After 15 min, 10 μl of 100 μg/ml PI was added in the dark on ice; after 5 min, the resulting mixture was again centrifuged at 1000 *g* for 5 min. The cells were suspended in 50 μl of 1× binding buffer. Images were captured using a fluorescence microscope and CellSens software. Apoptotic cells were observed in 200 randomly selected sample cells and 5 parallel samples at each time point. The cells were distinguished as normal cells (Hoechst 33,342+/Annexin V+), early apoptosis cells (Hoechst33342+/Annexin V++), and late apoptosis and necrosis cells (Annexin V++/PI++).

### Statistical analysis

All quantitative data were analysed by ANOVA in SPSS 19.0 (SPSS Inc., Chicago, Illinois, USA) and expressed as the means ± SE. A *p*-value of < 0.05 was considered significant.

## Results

### ATP and Bax-inhibitor increased *E. tenella* infection rates

**Fig. 1:**
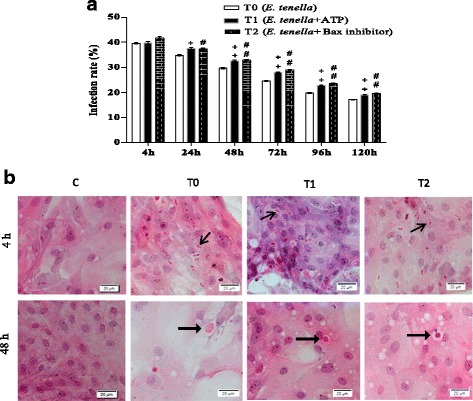
*E. tenella* infection rates. **a** Quantitative determination of *E. tenella* infection (*n* = 5). **b** Caecal epithelial cells in groups C, T0, T1, and T2 at 4, and 48 h, respectively. ^+^ *P* < 0.05 vs. T0, ^++^ *P* < 0.01 vs. T0; ^#^ *P* < 0.05 vs. T0, ^##^ *P* < 0.01 vs. T0, as indicated below the figures. “” represents sporozoites, “” represents trophozoites. Magnification 400×

The *E. tenella* infection rate was detected by HE staining (Fig. 1a-b; Additional file 1). The results indicated that although no significant difference in *E. tenella* infection rates was observed among T0, T1 and T2 groups at 4 h after infection by 4 × 10^5^ *E. tenella* sporozoites per chamber slide (*P* > 0.05), and groups T1 and T2 exhibited higher *E. tenella* infection rates than group T0 (*P* < 0.05 or *P* < 0.01) at 24, 48, 72, 96 and 120 h after infection.

### ATP and Bax-inhibitor decreased MPTP opening in *E. tenella* host cells

**Fig. 2:**
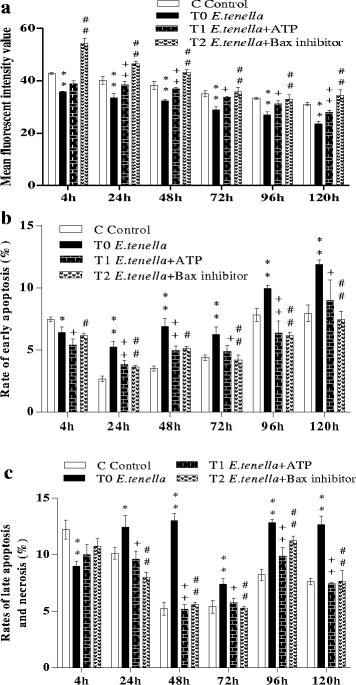
The influence of ATP and Bax on MPTP opening and the rate of early apoptosis, late apoptosis, and necrosis of *E. tenella* host cells. **a** Effect of ATP and Bax on MPTP opening of *E. tenella* host cells. **b** Effect of ATP, and Bax on the early apoptosis rate of *E. tenella* host cells. **c** Effect of ATP and Bax on the rate of late apoptosis, and necrosis of *E. tenella* host cells. ^*^ *P* < 0.05 vs. C, ^**^ *P* < 0.01 vs. C, the same as below figures

**Fig. 3:**
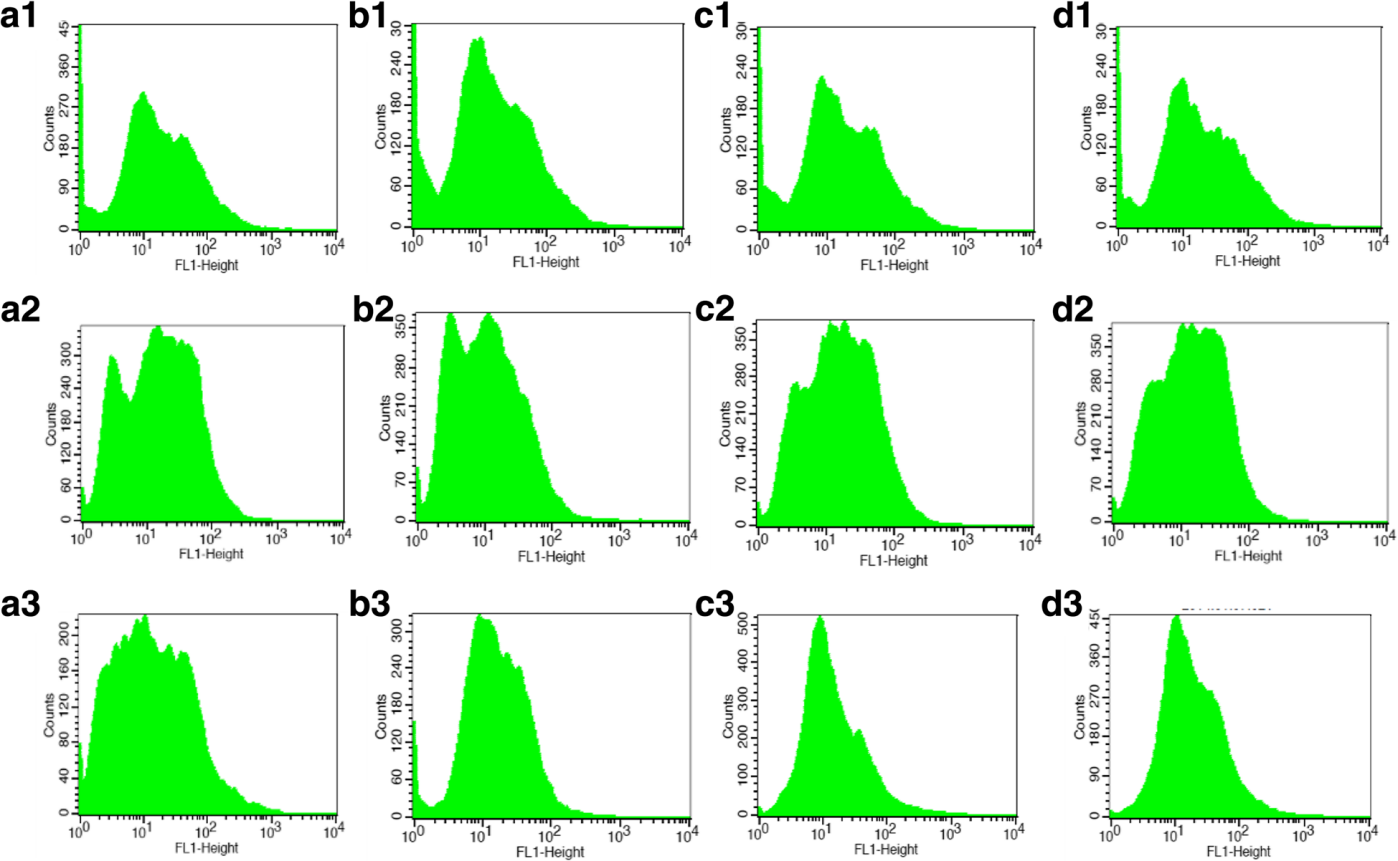
The dynamic detection of the MPTP opening in chick embryo caecal cells by flow cytometry. At 24, 72, and 120 h, **a**1–3, **b**1–3, **c**1–3, and **d**1–3 represent the changes of MPTP opening in groups C, T0, T1, and T2 respectively

A higher fluorescence value indicates a lower degree of MPTP opening. MPTP opening of the cells in groups C, T0, T1 and T2 were detected by FC. The results showed that MPTP opening of group T0 was higher (*P* < 0.01) than that of group C at 4, 24, 48, 72, 96, and 120 h (Figs. 2a and 3; Additional file 2). The results also indicated that MPTP opening of group T1 was visibly lower (*P* < 0.05 or *P* < 0.01) than that of group T0 (Figs. 2a and 3). MPTP opening of group T2 was also lower (*P* < 0.01) than that of group T0 (Figs. 2a and 3; Additional file 2).

### ATP and Bax-inhibitor decreased apoptosis rate of *E. tenella* host cells

**Fig. 4:**
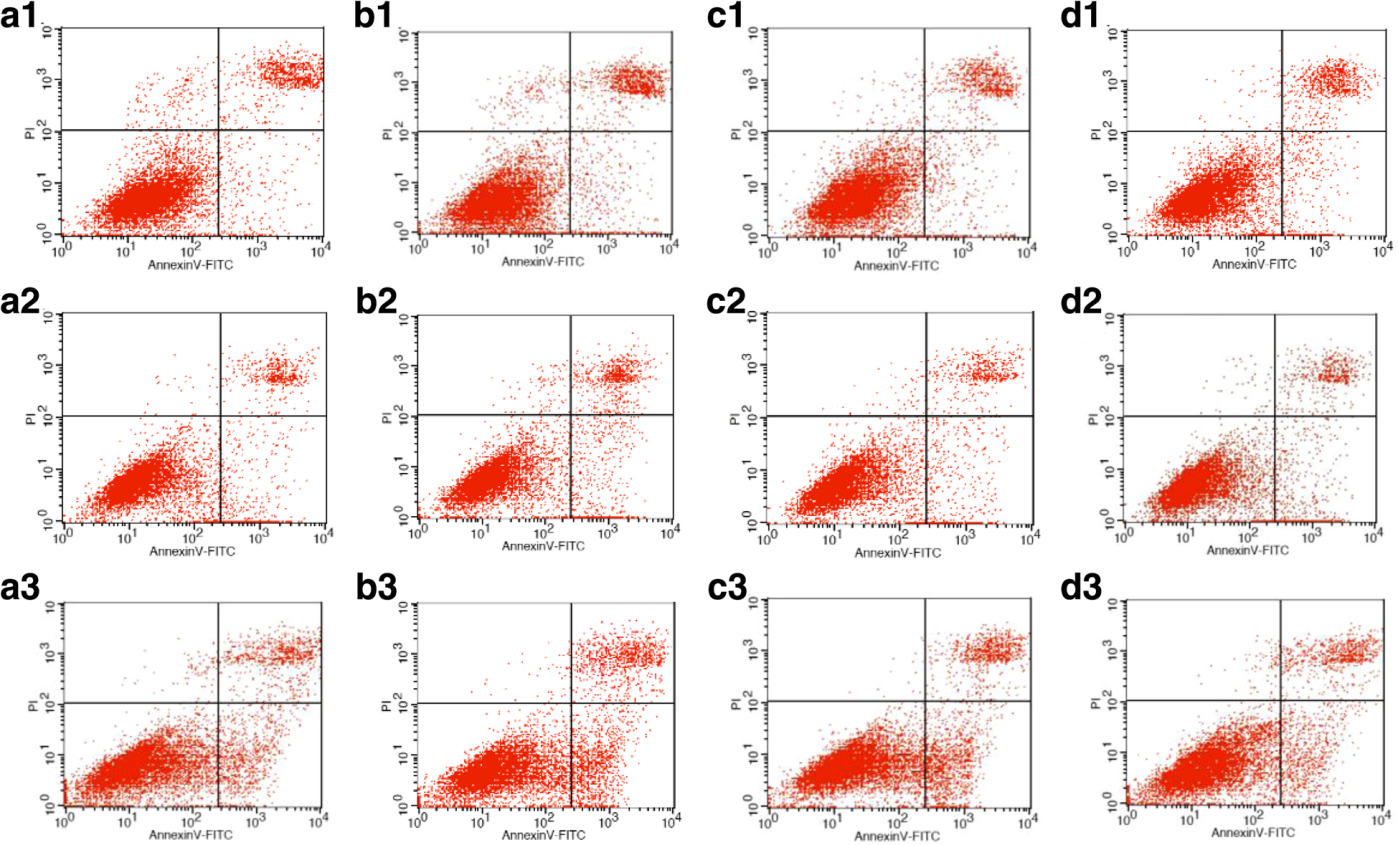
Annexin V/PI-based apoptosis detection in chick embryo caecal cells by flow cytometry. At 24, 72, and 120 h, **a**1–3, **b**1–3, **c**1–3, and **d**1–3 represent the rates of early apoptosis, late apoptosis and necrosis of groups C, T0, T1, and T2 respectively

**Fig. 5:**
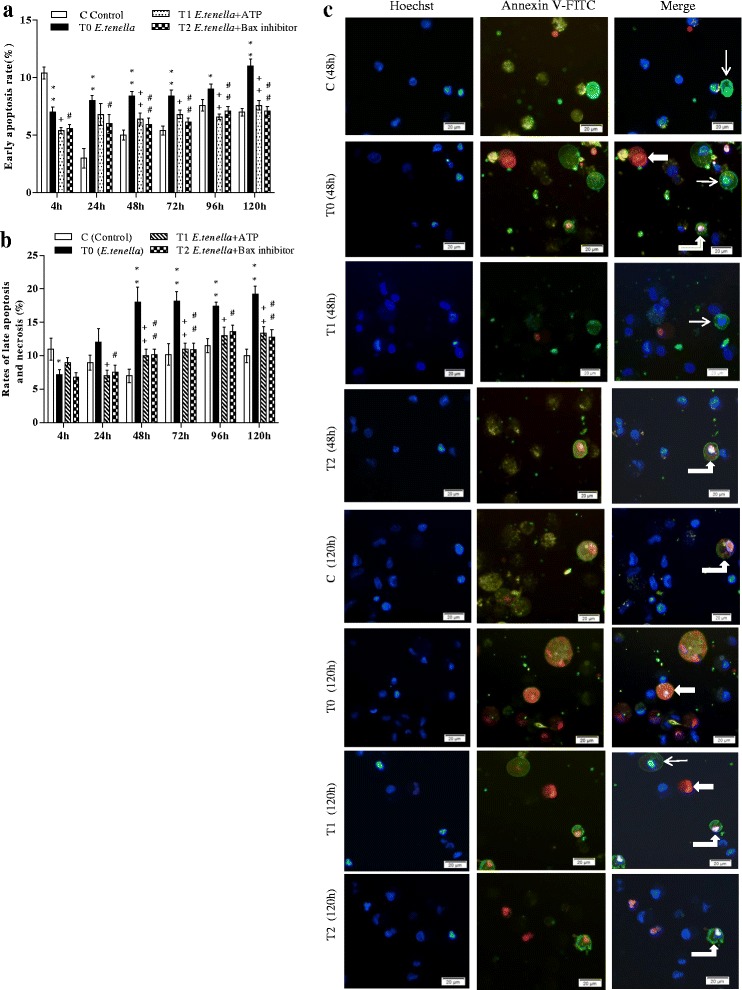
Hoechst-Annexin V/PI-based apoptosis detection in chick embryo caecal cells. **a** Quantitative determination of early apoptosis (n = 5). **b** Quantitative determination of late apoptosis and necrosis (n = 5). **c** Hoechst staining (blue) and Annexin V/PI staining (green/red) to detect apoptosis cells in group C, T0, T1 and T2 at 48 and 120 h, respectively; merge is Hoechst staining/Annexin V/PI staining overlay. “” represents early apoptosis cell, “” represents late apoptosis cell, “” represents necrosis cell. Magnification 400

Early apoptosis, late apoptosis and necrosis rates in groups C, T0, T1 and T2 were detected by FC and Hoechst-Annexin V-FITC-PI staining. The results of FC showed that 4 h after infection with *E. tenella*, the early apoptosis, late apoptosis and necrosis rates in group T0 were lower than those in group C, whereas the early apoptosis, late apoptosis and necrosis rates in group T0 were higher than those in group C to varying degrees at 24, 48, 72, 96 and 120 h (Figs. 2b-c, and 4; Additional files 3 and 4). The results of Hoechst-Annexin V-FITC-PI staining showed that the early apoptosis rate in group T0 significantly decreased at 4 h (*P* < 0.01) compared with that in group C. However, the early apoptosis rate in group T0 significantly increased at 24, 48, 72, 96 and 120 h (*P* < 0.05 or *P* < 0.01) compared with that in group C (Fig. 5a and c; Additional file 5). Compared with rates of late apoptosis and necrosis in group C, those in group T0 significantly decreased at 4 h (*P* < 0.05) and did not significantly differ at 24 h (*P* > 0.05) but highly significantly increased at 48, 72, 96 and 120 h (*P* < 0.05 or *P* < 0.01) (Fig. 5b and c; Additional file 6). Group T1 showed significantly lower or highly significant rates of early apoptosis, late apoptosis and necrosis than group T0 (Figs. 2b-c, 4, and 5a-c; Additional files 5 and 6). The rates of early apoptosis, late apoptosis and necrosis in group T2 were significantly lower or highly significantly lower compared to those in group T0 (Figs. 2b-c, and 4, and 5a-c; Additional files 5 and 6).

## Discussion

As an intracellular parasite, the infection rate of *E. tenella* was the basic condition of this experiment. The results showed that group T0 host cells featured high rates of *E. tenella* infection (Fig. 1a-b; Additional file 1), and the infection rates decreased with prolonged infection time, consistent with the results obtained in previous reports [6]. After treatment with ATP and Bax inhibitor, *E. tenella* infection rates remarkably increased, indicating the successful construction of experimental models.

After infection with *E. tenella*, the results of the two methods were consistent and showed that *E. tenella* inhibited host cell apoptosis during the early stages of *E. tenella* development and promoted host cell apoptosis during the middle and late developmental stages, supporting previous results [6, 24]. *E. tenella* also increased host-cell MPTP opening.

ATP plays a key role in maintaining normal cellular functions. The results by FC showed that ATP inhibited MPTP opening. The results of the two other methods used were consistent in this experiment, showing that ATP decreased the host cell apoptosis rate. ATP synthesis mainly depends on the mitochondrial membrane potential, and antioxidants can inhibit cell apoptosis by preventing decreases in ATP [25]. The exchange between ATP in mitochondria and ADP in the cytoplasm occurs via ANT. High concentrations of ATP can stabilise ANT in M constellation to inhibit MPTP opening, thus further preventing cell apoptosis [26]. Upon serious stimulation, MPTP of the cell irreversibly opens, uncoupling oxidative phosphorylation, inhibiting ATP synthesis, and promoting ATP decomposition, eventually leading to necrocytosis [27]. A decrease in ATP results from the exhaustion of the membrane potential gradient caused by MPTP opening, and the release of apoptosis factors occurs due to swelling and the fracture of mitochondria caused by MPTP opening [28, 29]. In the present study, after treatment with ATP, MPTP opening in host cells was significantly inhibited, and the apoptosis rate of host cells significantly decreased, showing that ATP is a key factor in regulating the apoptosis of *E. tenella* host cells. These results are consistent with those of previous studies [25, 26]. At 4 h after infection with *E. tenella*, MPTP opening was increased in group T0 cells, whereas the apoptosis rate in group T0 decreased (Fig. 2a-c; Additional file 2). This result was due to a notable increase in the amounts of nuclear factor-κB and Bcl-xl, which can inhibit host cell apoptosis during the early stages of *E. tenella* development [30]. The involvement of other factors in this process must be further studied.

Bax is an important pro-apoptotic protein in the Bcl-2 family. V5 is a Bax-inhibitor and can protect cells from Bax-mediated apoptosis. The results of FC showed that the Bax inhibitor inhibited MPTP opening. The results of the other two methods used were consistent in this experiment, showing that the Bax inhibitor decreased the host cell apoptosis rate. Bax may regulate the mechanism of cell apoptosis in the following manner: first, Bax mediates changes in mitochondria permeability, further causing the synthetic obstruction of ATP; subsequently, Bax changes cell oxidation-reduction; third, Bax releases relevant factors that activate the signalling pathways of the caspase family [31]. Bax is assumed to regulate mitochondrial membrane permeability in two ways: first, Bax directly regulates MPTP opening [32]; second, Bax forms pores in the mitochondrial membrane and regulates mitochondrial membrane permeability. Brenner reported that Bax can combine with ANT and form pores in an artificial lipid bilayer in vitro, although the combination of Bcl-2 and ANT can inhibit the formation of Bax channels, thus further inhibiting changes in mitochondrial membrane permeability [33]. Studies have demonstrated that the oligomerisation of Bax subfamily proteins is promoted by the interactions of BH-3 and Bax subfamily proteins, and these complexes enter mitochondria to release cytochrome C, leading to apoptosis [34]. During the early stages of infection, *Cryptosporidium* inhibits host cell apoptosis by promoting the expression of the anti-apoptotic protein Bcl-2 but decreasing the expression of the pro-apoptotic protein Bax and reducing the release of apoptosis-related mitochondrial proteins. Conversely, during the late stages of infection, the development of polypide can inhibit Bcl-2 expression and increase Bax expression, which promotes MPTP opening and further induces cell apoptosis [35]. The results of the present study demonstrated that *E. tenella* promotes MPTP opening and further increases the cell apoptosis rate. However, V5 significantly decreases the cell apoptosis rate and inhibits MPTP opening, showing that Bax is a key regulator of apoptosis in *E. tenella* host cells. These results are consistent with those reported for other pathogens [35].

## Conclusions

In conclusion, ATP and Bax inhibitors inhibited the MPTP opening and decreased the rate of early apoptosis, late apoptosis, and necrosis of *E. tenella* host cells. This finding shows that ATP and Bax play important roles in regulating the apoptosis of *E. tenella* host cells.

## Additional files

Additional file 1: *E. tenella* infection rates. ^+^ *P* < 0.05 vs. T0, ^++^ *P* < 0.01 vs. T0; ^#^P < 0.05 vs. T0, ^##^P < 0.01 vs. T0, as indicated below the figures. (DOCX 14 kb)

Additional file 2: The influence of ATP and Bax on MPTP opening of *E. tenella* host cells by flow cytometry. ^*^P < 0.05 vs. C, ^**^P < 0.01 vs. C, the same as below figures. (DOCX 14 kb)

Additional file 3: The influence of ATP and Bax on the rate of early apoptosis of *E. tenella* host cells by flow cytometry. (DOCX 14 kb)

Additional file 4: The influence of ATP and Bax on the late apoptosis, and necrosis of *E. tenella* host cells by flow cytometry. (DOCX 14 kb)

Additional file 5: The influence of ATP and Bax on the rate of early apoptosis of *E. tenella* host cells by Hoechst-Annexin V/PI-based apoptosis detection. (DOCX 14 kb)

Additional file 6: The influence of ATP and Bax on the late apoptosis, and necrosis of *E. tenella* host cells by Hoechst-Annexin V/PI-based apoptosis detection. (DOCX 14 kb)

## Abbreviations

Annexin V-FITC: Fluorescein isothiocyanate-conjugated Annexin V
ANT: Adenine nucleotide transporter
ATP: Adenosine triphosphate
BW: Body weight
CsA: Cyclosporin A
*E. tenella*: *Eimeria tenella*
FC: Flow cytometry
HE: Haematoxylin and eosin Stain
MPTP: Mitochondrial permeability transition pore
PI: Propidium iodide
SPF: Specific pathogen-free
V5: H-VPMLK-OH, Bax-Inhibiting Peptide
VDAC: Voltage-dependent anion channel
