# Supplementary material for: Effect of ATP and Bax on the apoptosis of Eimeria tenella host cells
Source: BMC Vet Res. 2017 Dec 28;13:399. doi: 10.1186/s12917-017-1313-z (PMC5745796; doi:10.1186/s12917-017-1313-z)
Supplement: Supplementary file 1 — E. tenella infection rates. + P < 0.05 vs. T0, ++ P < 0.01 vs. T0; #P < 0.05 vs. T0, ##P < 0.01 vs. T0, as indicated below the figures. (DOCX 14 kb) [file 12917_2017_1313_MOESM1_ESM.docx]

**Additional file 1**

*E. tenella* infection rates.

| Time | T0 | T1 | T2 |
| --- | --- | --- | --- |
| 4h | 39.62±0.37 | 39.80±0.43 | 41.80±0.54 |
| 24h | 34.80±0.36 | 37.50±0.40+ | 37.40±0.37# |
| 48h | 29.70±0.28 | 32.60±0.31++ | 33.00±0.18## |
| 72h | 24.61±0.19 | 27.90±0.17++ | 29.00±0.21## |
| 96h | 19.80±0.20 | 22.70±0.22++ | 23.60±0.16## |
| 120h | 17.10±0.19 | 18.90±0.26++ | 19.60±0.23## |
