# Supplementary material for: Effect of ATP and Bax on the apoptosis of Eimeria tenella host cells
Source: BMC Vet Res. 2017 Dec 28;13:399. doi: 10.1186/s12917-017-1313-z (PMC5745796; doi:10.1186/s12917-017-1313-z)
Supplement: Supplementary file 2 — The influence of ATP and Bax on MPTP opening of E. tenella host cells by flow cytometry. *P < 0.05 vs. C, **P < 0.01 vs. C, the same as below figures. (DOCX 14 kb) [file 12917_2017_1313_MOESM2_ESM.docx]

**Additional file 2**

The influence of ATP and Bax on MPTP opening of *E. tenella* host cells by flow cytometry.

| Time | C | T0 | T1 | T2 |
| --- | --- | --- | --- | --- |
| 4h | 42.90±0.43 | 35.75±0.27** | 38.89±1.03 | 54.27±2.08## |
| 24h | 40.17±1.50 | 33.43±1.81** | 38.50±1.27++ | 46.58±0.88## |
| 48h | 38.23±1.57 | 32.31±0.65** | 36.90±0.63++ | 43.27±1.02## |
| 72h | 34.99±1.16 | 28.97±1.07** | 33.68±0.49+ | 35.73±1.82## |
| 96h | 33.32±0.38 | 26.99±1.14** | 31.28±1.15+ | 32.98±1.60## |
| 120h | 31.15±0.62 | 23.62±0.94** | 28.05±0.70++ | 34.43±2.14## |
