# Supplementary material for: Effect of ATP and Bax on the apoptosis of Eimeria tenella host cells
Source: BMC Vet Res. 2017 Dec 28;13:399. doi: 10.1186/s12917-017-1313-z (PMC5745796; doi:10.1186/s12917-017-1313-z)
Supplement: Supplementary file 3 — The influence of ATP and Bax on the rate of early apoptosis of E. tenella host cells by flow cytometry. (DOCX 14 kb) [file 12917_2017_1313_MOESM3_ESM.docx]

**Additional file 3**

The influence of ATP and Bax on the rate of early apoptosis of *E. tenella* host cells by flow cytometry.

| Time | C | T0 | T1 | T2 |
| --- | --- | --- | --- | --- |
| 4h | 7.47±0.19 | 6.41±0.45* | 5.40±0.50+ | 6.15±0.19# |
| 24h | 2.67±0.22 | 5.25±0.43** | 3.83±0.31++ | 3.64±0.13## |
| 48h | 3.50±0.20 | 6.88±0.68** | 4.96±0.34++ | 5.11±0.18# |
| 72h | 4.38±0.21 | 6.27±0.58** | 4.88±0.46+ | 4.19±0.42## |
| 96h | 7.82±0.53 | 9.97±0.24** | 6.37±0.99++ | 6.18±0.21## |
| 120h | 7.96±0.66 | 11.90±0.36** | 9.00±1.63+ | 7.47±0.65## |
