# Supplementary material for: Effect of ATP and Bax on the apoptosis of Eimeria tenella host cells
Source: BMC Vet Res. 2017 Dec 28;13:399. doi: 10.1186/s12917-017-1313-z (PMC5745796; doi:10.1186/s12917-017-1313-z)
Supplement: Supplementary file 4 — The influence of ATP and Bax on the late apoptosis, and necrosis of E. tenella host cells by flow cytometry. (DOCX 14 kb) [file 12917_2017_1313_MOESM4_ESM.docx]

**Additional file 4**

The influence of ATP and Bax on the late apoptosis, and necrosis of *E. tenella* host cells by flow cytometry.

| Time | C | T0 | T1 | T2 |
| --- | --- | --- | --- | --- |
| 4h | 12.21±0.84 | 9.00±0.44** | 10.00±0.88 | 10.78±0.67 |
| 24h | 10.11±0.52 | 12.45±1.00* | 9.60±0.73+ | 8.01±0.46## |
| 48h | 5.23±0.58 | 13.02±0.63** | 5.18±0.43++ | 5.61±0.19## |
| 72h | 5.40±0.55 | 7.39±0.48** | 5.78±0.36+ | 5.29±0.13## |
| 96h | 8.23±0.51 | 12.83±0.26** | 9.90±0.72++ | 11.25±0.40## |
| 120h | 7.64±0.30 | 12.68±0.73** | 7.46±0.06++ | 7.68±0.92## |
