# Supplementary material for: Effect of ATP and Bax on the apoptosis of Eimeria tenella host cells
Source: BMC Vet Res. 2017 Dec 28;13:399. doi: 10.1186/s12917-017-1313-z (PMC5745796; doi:10.1186/s12917-017-1313-z)
Supplement: Supplementary file 5 — The influence of ATP and Bax on the rate of early apoptosis of E. tenella host cells by Hoechst-Annexin V/PI-based apoptosis detection. (DOCX 14 kb) [file 12917_2017_1313_MOESM5_ESM.docx]

**Additional file 5**

The influence of ATP and Bax on the rate of early apoptosis of *E. tenella* host cells by Hoechst-Annexin V/PI-based apoptosis detection.

| Time | C | T0 | T1 | T2 |
| --- | --- | --- | --- | --- |
| 4h | 10.40±0.51 | 7.00±0.45** | 5.40±0.24+ | 5.60±0.32# |
| 24h | 3.00±0.84 | 8.00±0.45** | 6.80±0.97 | 6.00±0.78# |
| 48h | 5.00±0.45 | 8.40±0.40** | 6.40±0.51++ | 5.95±0.53## |
| 72h | 5.40±0.40 | 8.40±0.51** | 6.80±0.37+ | 6.15±0.34## |
| 96h | 7.60±0.51 | 9.00±0.45* | 6.60±0.24++ | 7.10±0.38## |
| 120h | 7.00±0.32 | 11.00±0.63** | 7.60±0.40++ | 7.08±0.41## |
