# Supplementary material for: Effect of ATP and Bax on the apoptosis of Eimeria tenella host cells
Source: BMC Vet Res. 2017 Dec 28;13:399. doi: 10.1186/s12917-017-1313-z (PMC5745796; doi:10.1186/s12917-017-1313-z)
Supplement: Supplementary file 6 — The influence of ATP and Bax on the late apoptosis, and necrosis of E. tenella host cells by Hoechst-Annexin V/PI-based apoptosis detection. (DOCX 14 kb) [file 12917_2017_1313_MOESM6_ESM.docx]

**Additional file 6**

The influence of ATP and Bax on the late apoptosis, and necrosis of *E. tenella* host cells by Hoechst-Annexin V/PI-based apoptosis detection.

| Time | C | T0 | T1 | T2 |
| --- | --- | --- | --- | --- |
| 4h | 11.00±1.67 | 7.20±0.71* | 9.00±0.71 | 6.80±0.69 |
| 24h | 9.00±1.14 | 12.00±2.10 | 7.00±0.89+ | 7.60±0.99# |
| 48h | 7.00±1.00 | 18.00±2.28** | 10.00±1.00++ | 10.20±0.83## |
| 72h | 10.20±1.60 | 18.20±1.39** | 11.00±0.91++ | 10.90±1.02## |
| 96h | 11.50±1.05 | 17.40±0.60** | 13.00±1.26+ | 13.60±0.98# |
| 120h | 10.00±0.982 | 19.20±1.20** | 13.40±0.93++ | 12.80±1.09## |
